# Colorectal Cancer Screening Preferences of Recipients and Providers: A Dual‐Perspective Discrete Choice Experiment

**DOI:** 10.1002/cam4.71341

**Published:** 2025-11-02

**Authors:** Weimin Guan, Nan Zhang, Zhengyang Lu, Mingjun Zhang, Yan Liu, Pengfei Li, Hang Yu, Boyu Liu, Wenxuan Yan, Guifeng Ma, Youhua Lu

**Affiliations:** ^1^ School of Public Health Shandong Second Medical University Weifang Shandong China; ^2^ Shandong Cancer Hospital and Institute, Shandong First Medical University and Shandong Academy of Medical Sciences Jinan Shandong China; ^3^ School of Public Health and Health Management Shandong First Medical University Jinan Shandong China

**Keywords:** CRC, preference, Providers, Recipients, screening

## Abstract

**Background:**

The low participation rate in colorectal cancer (CRC) screening may be partly attributed to the lack of consideration for the preferences of both Recipients and Providers. This study aims to explore these preferences to inform the optimization of screening design and the improvement of implementation strategies.

**Methods:**

A discrete choice experiment (DCE) was conducted in Shandong Province to examine CRC screening preferences of Recipients and Providers. The attributes and levels of the DCE were determined using a systematic literature review and explored qualitatively. Questionnaires were generated through a partial factor design, and used a mixed logit model to analyze the data. Relative importance scores (RIS) and marginal willingness to pay were used to quantify preferences, and probability density functions were employed to predict changes in participation rates under varying attribute levels.

**Results:**

Preference data from 570 Recipients and 532 Providers were analyzed. The DCE included five attributes: screening cost (four levels), screening interval (four levels), bowel preparation (two levels), screening accuracy (three levels), and reduction in CRC‐related mortality risk (three levels). All attributes significantly influenced preferences. The RIS indicated that Recipients prioritized screening cost (42.8%), followed by interval (24.3%), mortality risk reduction (16.2%), accuracy (10.7%), and bowel preparation (6.0%), whereas Providers emphasized bowel preparation (35.4%), interval (31.7%), cost (25.1%), mortality risk reduction (6.4%), and accuracy (1.3%). Both groups showed strong support for biennial screening. Shortening the interval from 10 to 2 years increased Recipients' willingness to pay by CNY 1052.95 and Providers' expected charge by CNY 1370.84, which was also associated with higher predicted participation rates.

**Conclusion:**

Recipients and Providers differed in the degree of preference for the five CRC screening attributes, but the directions of their preferences were consistent. Therefore, screening strategies should aim to balance the perspectives of both groups. Where feasible, a biennial screening program that includes bowel preparation, minimizes costs and mortality risk, and maximizes accuracy is recommended.

## Abstract 2

Preferences for colorectal cancer (CRC) screening differ between Recipients and Providers. Therefore, screening strategies should be designed to balance the interests of both groups. Where feasible, a biennial screening program that includes bowel preparation, minimizes costs and mortality risk, and maximizes accuracy is recommended.

## 1 Introduction

Colorectal cancer (CRC) is one of the most common malignancies worldwide, with both its incidence and mortality showing a steadily increasing trend, imposing a substantial disease burden. It is estimated that approximately 1.9 million new CRC cases are diagnosed globally each year, ranking third among all cancers [1], while CRC‐related deaths reach about 935,000 annually, ranking second in cancer mortality [2, 3]. As the largest developing country, China faces an especially severe CRC burden. According to GLOBOCAN data, by 2020, there were approximately 555,000 newly diagnosed CRC cases in China, accounting for 28.8% of all new CRC cases worldwide, and around 286,000 CRC‐related deaths, representing 30.6% of global CRC mortality [4]. Effective CRC screening is vital for reducing this burden and associated mortality risk [1, 5, 6, 7]. Studies have shown that by 2020, the global adherence rate to CRC screening was approximately 54% [8], with compliance rates close to 50% in the United States, an average of 55% in Canada, and ranging from 19% to 69% across Europe [9]. In China, there are currently no surveillance data or medical records available to accurately assess screening adherence; however, screening coverage has been steadily increasing [10]. Shandong Province, as one of the key pilot regions of the National Cancer Early Detection and Treatment Program, has implemented large‐scale early detection and treatment projects targeting rural areas, the Huaihe River Basin, and urban populations. In regions where these programs were initiated earlier, a declining trend in both incidence and mortality of related cancers has already been observed. Furthermore, in 2025, “CRC screening for high‐risk populations” has been included in Shandong's list of 20 key livelihood initiatives, aiming to provide 200,000 free CRC screening tests across the province. Although public attitudes toward CRC screening are generally positive, participation rates remain low [11, 12, 13, 14, 15].

Low screening uptake is influenced by multiple factors, including “demand‐side” elements such as socioeconomic status, health literacy, culture, and religious beliefs [16], as well as “supply‐side” factors like policy environment, screening service models [17, 18], and accessibility of screening resources. Evidence suggests that Recipients' adherence to screening is influenced by their knowledge and preferences. Although recommendations from Providers can improve adherence, Recipients may still prefer screening modalities different from those recommended [19]. For instance, in CRC screening, Providers often favor colonoscopy; however, offering colonoscopy as the sole option may reduce adherence among Recipients. In the healthcare service market, Providers' behavior typically exhibits externalities, as they not only consider their own interests but also take into account the health benefits of patients [20, 21]. The American College of Physicians recommends that clinicians engage patients in shared decision‐making about CRC screening tests, considering benefits, risks, costs, availability, frequency, and patient values and preferences. To maximize the effectiveness of screening, policymakers should give full and priority consideration to Recipients' preferences, while also taking Providers' preferences into account. Therefore, understanding the preferences of both Recipients and Providers is essential to improving screening participation [17, 22, 23]. Existing studies predominantly focus on the Recipients perspective, often neglecting the interactions and differences in preferences between both parties [24, 25], which limits comprehensive and precise evidence to optimize screening strategies.

Discrete choice experiments (DCEs) are a research method widely used to assess individual preferences in cancer screening [26]. This approach simulates hypothetical scenarios to reveal respondents' underlying preferences, providing decision‐makers with evidence to develop more cost‐effective and personalized screening strategies.

To optimize screening strategies and effectively improve adherence, this study builds on a dual supply–demand perspective and employs DCEs to systematically assess the genuine needs and preferences of both Recipients and Providers in CRC screening [19]. It quantitatively analyzes their marginal willingness to pay and to provide for different screening attributes and levels, and predicts changes in participation rates. The findings help identify similarities and differences in screening preferences between both parties, offering theoretical support and policy recommendations for designing more precise, accessible, and acceptable CRC screening strategies.

## 2 Materials and Methods

### 2.1 Discrete Choice Experiment

The discrete choice experiment (DCE) is a stated preference method based on random utility theory and has been widely used in cancer screening research [27]. A DCE asks respondents to select their preferred option from a set of hypothetical alternatives, each defined by different attributes and levels, in order to identify the key factors that influence screening decisions. Following the 10 recommended standards for stated preference studies proposed by the International Society for Pharmacoeconomics and Outcomes Research (ISPOR) in 2011, this study conducted a DCE to assess the screening preferences of both Recipients and Providers for CRC [19, 28, 29]. The design and implementation are detailed as follows:

### 2.2 Attributes and Levels

Between January and February 2025, attributes and levels were identified through a systematic qualitative approach. First, a comprehensive search of CNKI, Web of Science, and PubMed yielded 19 attributes related to CRC screening (levels were not counted as the same attribute had varying levels across studies; Table S1). Second, the 10 most frequently reported attributes were selected to construct a consultation list (Table S2). Twelve experts (Table S3) ranked the importance of these attributes and provided suggestions for refinement. Subsequently, two focus group discussions were conducted with community residents (six participants each in Tai'an and Jining, *n* = 12), who were asked to rank attribute importance. Based on these findings, five attributes and their corresponding levels were finalized for the DCE [30, 31]. Details are presented in Table 1.

**TABLE 1:** Definitions of attributes and their levels.

Attribute	Definition	Level
Screening costs	The cost charged by healthcare Providerss or paid out‐of‐pocket by individuals during the colorectal cancer screening process	0￥
		100￥
		500￥
		1000￥
Bowel preparation	The process of cleansing the bowel of feces and residual contents through a series of preparatory measures prior to colorectal cancer screening	Yes
		No
Screening interval	The interval between two consecutive screening tests	Annually
		Every 2 years
		Every 5 years
		Every 10 years
Screening accuracy	The ability of screening to detect colorectal cancer and its precancerous lesions	50%
		70%
		90%
Reduction in CRC‐related mortality	Colorectal cancer screening facilitates the early detection of colorectal cancer or precancerous lesions, enabling timely and effective interventions that can reduce the risk of mortality associated with colorectal cancer	10%
		50%
		90%

*Note:* (1) Screening costs (Recipients perspective): The portion of the screening cost that is not covered by health insurance or government subsidies and must be borne by the individual. (2) Screening costs (Providers perspective): The fee charged by healthcare providers during the implementation of colorectal cancer screening.

### 2.3 Experimental Design

We employed SAS macros to conduct a fractional factorial design to enhance the efficiency and precision of the study design [32]. To ensure orthogonality, level balance, and minimal overlap, a D‐efficiency design was generated, producing 12 choice sets that were randomly allocated into two questionnaire versions (Block = 2). Each version included one repeated choice set (Set 2 = Set 7) to test respondents' comprehension of the questionnaire (internal consistency check; Tables S4 and S5) [33]. In total, each version contained seven choice sets, with the repeated set excluded from the final analysis. Each choice set included two hypothetical screening alternatives and one opt‐out option (i.e., “In real life, would you be willing to provide or participate in the screening as described?”).

### 2.4 Survey Instrument

The paper‐based questionnaire administered to respondents comprised two sections (Data S1): demographic characteristics (age, sex, educational attainment, marital status, family history of cancer, region, and professional title [Providers‐specific]) and a series of DCE choice sets accompanied by a self‐assessment question on task comprehension. Trained interviewers provided one‐on‐one guidance to explain the survey and answer queries, without influencing respondents' choices. All participants provided written informed consent prior to participation. Ethical approval was obtained from the Ethics Committee of the Affiliated Cancer Hospital of Shandong First Medical University (Approval No.: SDTHEC2024003167).

### 2.5 Pilot Study

Between February 23 and 28, 2025, a pilot study was conducted in Weifang to assess the appropriateness of the selected attributes and levels, the validity of the questionnaire, and respondents' comprehension and acceptability. A total of 46 Recipients and 54 Providers were surveyed. Eligibility criteria were as follows: (1) community residents aged 40–74 years; (2) healthcare Providers involved in CRC screening, including those from the cancer center office, health examination center, endoscopy, pathology, laboratory, and electrocardiography departments; (3) no major organ dysfunction or psychiatric disorders; and (4) voluntary participation with the ability to complete the questionnaire. The exclusion criterion, applied to both Recipients and Providers, was the inability to understand the purpose and content of the experiment or to make trade‐off decisions even after detailed explanation by the investigator. Data from the pilot study were not included in the main analysis. It is worth mentioning that in order to verify whether the respondents could correctly understand, handle and answer the multiple‐choice questions, we used the cognitive interview method in the determination of attributes and levels as well as in the pilot research stage. Through this method, the scientificity and effectiveness of the questionnaire can be significantly improved [34, 35].

### 2.6 Sample Size Estimation

The minimum sample size required for this study was calculated based on the widely accepted rule of thumb [27], expressed as: $n>\frac{500*c}{t*a}$, where 500 is a constant, *c* is the maximum number of levels for any attribute, *a* is the number of alternatives per choice set in the DCE, and *t* is the number of choice sets per questionnaire version. According to this formula, the minimum sample size needed was 286 participants.

### 2.7 Study Population and Data Collection

Based on the pilot study, the questionnaire was revised as follows: (1) removal of nonessential questions to reduce respondent burden; (2) reordering of selected items to enhance logical flow and coherence; and (3) reduction in the number of DCE choice tasks to mitigate cognitive fatigue. From February to April 2025, a cross‐sectional survey was conducted in Shandong Province, one of the key pilot provinces of the National Program for Early Diagnosis and Treatment of Cancer. Respondents were randomly recruited in Weifang (eastern region), Tai'an (central region), and Jining (western region), representing different geographic locations and levels of economic development, and were asked to complete a self‐administered paper questionnaire. Upon completion, participants received a small gift as appreciation. The inclusion and exclusion criteria were consistent with those of the pilot study.

### 2.8 Statistical Analyses

For each respondent, 18 observations were generated and converted into 18 rows (6 choice sets × 3 alternatives) of data in the format required for DCE analysis. Except for screening cost, all attribute coefficients were treated as random parameters and included as categorical variables, coded using dummy variables (each level represented as a binary variable, 1 = presence, 0 = otherwise, with one level serving as the reference category), which is the standard approach in DCE analyses [36]. The coefficient for screening cost was specified as fixed to facilitate the estimation of willingness to pay.

Within the framework of random utility theory, DCE data were analyzed using a mixed logit model, assuming normally distributed random parameters. Model estimation was conducted via simulated maximum likelihood with 200 Halton draws for Recipients and 500 for Providers. The study adhered to the DIRECT checklist (Table S6) [37].

In this study, it was assumed that each respondent would choose the screening option with the highest utility. The utility function was specified as follows:

$$\begin{array}{cc} U_{\mathrm{ni}}= & \beta_{0}+\beta_{1}\left(\text{Bowel preparation}:\mathrm{Yes}\right)+\beta_{2}\left(\text{Screening accuracy}:50\%\right)\\ & +\beta_{3}\left(\text{Screening accuracy}:70\%\right)+\beta_{4}\left(\text{Screening interval}:\text{Annually}\right)\\ & +\beta_{5}\left(\text{Screening interval}:\text{Every}\;\mathrm{two}\;\text{years}\right)\\ & +\beta_{6}\left(\;\text{Screening interval}:\text{Every five years}\right)\\ & +\beta_{7}\left(\text{Reduction in}\;\mathrm{CRC}-\text{related mortality}:10\%\right)\\ & +\beta_{8}\left(\text{Reduction in}\;\mathrm{CRC}-\text{related mortality}:50\%\right)\\ & +\beta_{9}\left(\text{Screening costs}\right)+\varepsilon . \end{array}$$

where $U_{\mathrm{ni}}$ denotes the utility derived by respondent 𝑛 from choosing alternative 𝑖; $\beta_{0}$ is the coefficient of the alternative‐specific constant, reflecting the mean preference toward CRC screening in the utility function; and $\beta_{1}-\beta_{9}$ represents the coefficients of the attribute levels, capturing the relative weights assigned to each attribute level.

The relative importance score (RIS) reflects the contribution of each attribute to decision‐making relative to others, calculated as the ratio of the maximum utility of an attribute to the sum of maximum utilities of all attributes.

$${\mathrm{RIS}}_{X}=\frac{U_{\text{Xmax}}}{U_{\text{total}}}$$

where *X* denotes a given attribute; *X* $U_{\text{Xmax}}$ represents the maximum utility of attribute; and $U_{\text{total}}$ is the total utility across all attributes.

Willingness to pay (WTP) quantifies the amount a respondent is willing to pay or accept as compensation for changes in CRC screening attributes and levels. In our study, the WTP was estimated in the preference space, this approach allows us to directly interpret WTP as the additional out‐of‐pocket cost that recipients are willing to pay, or the compensation that providers expect to receive, for a change in CRC screening attributes. It can be estimated using the following formula, where $\beta_{X}$ is the coefficient of any attribute level and $\beta_{\text{cost}}$ is the coefficient of screening cost:

$$\mathrm{WTP}=-\frac{\beta_{X}}{\beta_{\text{cost}}}$$

To estimate the expected uptake rate of the screening program when attribute levels change, a probability density function was used to model the variation in respondent participation resulting from changes in a given attribute level:

$$\mathrm{Pi}=\frac{\exp^{\beta X_{i}}}{\sum \exp^{\beta X_{j}}}$$

Here, *X* represents the vector of attribute level coefficients, and *β* denotes the regression coefficients for attributes and levels. Note: In calculating participation rates, we assumed only the baseline and target levels (or combinations), without considering the opt‐out option.

All statistical analyses were performed using *Stata* 17.0, and graphical visualizations were generated with *R* 4.5.0.

## 3 Results

A total of 1147 complete questionnaires were collected for the DCE survey. After excluding 45 that failed the consistency check, preference data from 570 recipients and 532 providers were included in the final analysis (see Tables S4 and S5 for details of the consistency check). Sociodemographic details are presented in Table 2.

**TABLE 2:** Basic demographic characteristics of the study population.

Demographic characteristics	Recipients perspective, *n* (%)	Providers perspective, *n* (%)
Total sample size	570	532
Age	59 (64–52)	36 (43–31)
Gender		
Male	247 (43.33)	148 (27.82)
Female	323 (56.67)	384 (72.18)
Education level		
Below undergraduate	532 (93.33)	71 (13.35)
Undergraduate and above	38 (6.67)	461 (86.65)
Marital status		
Married	531 (93.16)	456 (85.71)
Unmarried, divorced, widowed, or separated	39 (6.84)	76 (14.29)
Family history of cancer		
Yes	152 (26.67)	260 (48.87)
No	418 (73.33)	272 (51.13)
Region		
Eastern region	199 (34.91)	200 (37.59)
Central region	177 (31.05)	195 (36.65)
Western region	194 (34.04)	137 (25.75)
Professional title^a^		
None	N/A	60 (11.28)
Junior professional title		158 (29.70)
Intermediate professional title		223 (41.92)
Associate senior title or above		91 (17.11)

*Note:* Family history refers to whether any blood relatives — including parents, grandparents, siblings, uncles, aunts, and first cousins—have ever been diagnosed with cancer.

^a^ Unique to the supply‐side perspective.

### 3.1 Estimation of Aggregate Preferences and Attribute Relative Importance

The analysis of the Recipients' preference model indicated statistically significant differences for all attributes and levels except a 10% reduction in CRC‐related mortality (*β* = 0.047, *p* > 0.05). Ranked by relative importance, Recipients showed strong preferences for lower screening costs (*β* = −0.001, *p* < 0.001), shorter screening intervals (annually: *β* = 1.356, *p* < 0.001; every 2 years: *β* = 1.573, *p* < 0.001; every 5 years: *β* = 0.725, *p* < 0.001), greater reduction in CRC‐related mortality (50%: *β* = −0.519, *p* < 0.001), higher screening accuracy (50%: *β* = −1.345, *p* < 0.001; 70%: *β* = −0.970, *p* < 0.001), and the inclusion of bowel preparation (*β* = 0.210, *p* < 0.05).

The analysis of the Providers' preference model revealed statistically significant differences across all attributes and levels except for annual (*β* = 0.062, *p* > 0.05) and 5‐year (*β* = 0.169, *p* > 0.05) screening intervals. Ranked by relative importance, Providers showed stronger preferences for offering screening with bowel preparation (*β* = 0.812, *p* < 0.001), a 2‐year screening interval (*β* = 0.791, *p* < 0.001), charging lower screening costs (*β* = −0.001, *p* < 0.001), greater reduction in CRC‐related mortality (10%: *β* = −0.774, *p* < 0.001; 50%: *β* = −0.920, *p* < 0.001), and higher screening accuracy (50%: *β* = −1.054, *p* < 0.001; 70%: *β* = −1.024, *p* < 0.001). Details are shown in Table 3.

**TABLE 3:** Preference estimates and relative importance of attributes.

Attributes and levels	Ref.	Recipients perspective		Providers perspective	
		*β*/SD (95% CI)	RIS (%) (95% CI)	*β*/SD (95% CI)	RIS (%) (95% CI)
Bowel preparation					
Yes	No	0.210*/0.089 (0.036, 0.385)	6.0* (5^a^) (1.19, 10.86)	0.812***/0.087 (0.641, 0.982)	35.4*** (1) (27.66, 43.09)
Screening accuracy					
50%	90%	−1.345***/0.122 (−1.585, −1.106)	10.7** (4) (4.23, 17.26)	−1.054***/0.106 (−1.262, −0.847)	1.3 (5) (−6.52, 9.16)
70%		−0.970***/0.112 (−1.191, −0.750)		−1.024***/0.092 (−1.203, −0.844)	
Screening interval					
Annually	Every 10 years	1.356***/0.137 (1.088, 1.624)	24.3*** (2) (17.37, 31.16)	0.062/0.109 (−0.153, 0.276)	31.8*** (2) (22.93, 40.62)
Every 2 years		1.573***/0.133 (1.312, 1.833)		0.791***/0.111 (0.574, 1.007)	
Every 5 years		0.725***/0.117 (0.496, 0.955)		0.169/0.098 (−0.024, 0.362)	
Reduction in CRC‐related mortality					
10%	90%	0.047/0.111 (−0.170, 0.264)	16.2*** (3) (10.10, 22.30)	−0.774***/0.105 (−0.980, −0.567)	6.4 (4) (−1.99, 14.77)
50%		−0.519***/0.114 (−0.742, −0.296)		−0.920***/0.104 (−1.124, −0.716)	
Screening costs		−0.001***/0.000 (−0.002, −0.001)	42.8*** (1) (36.42, 49.11)	−0.001***/0.000 (−0.001, −0.000)	25.1*** (3) (18.39, 31.89)
ASC (opt‐out option)		−1.917***/0.136 (−2.183, −1.652)		−3.450***/0.156 (−3.756, −3.145)	

*Note:* (1) ASC (opt‐out option): Represents a specific constant term; the ASC refers to the opt‐out option, which was set as the reference alternative. (2) SD is the standard error of *β*.

^a^ The order of relative importance of the attributes as determined by the mixed logit model.

*p* < 0.05 is considered statistically significant (*p < 0.05; **p < 0.01; ***p < 0.001).

### 3.2 Preference Heterogeneity Analysis

Significant preference heterogeneity was observed among Recipients for bowel preparation, screening accuracy, screening interval, and reduction in CRC‐related mortality (*p* < 0.001). Among Providers, preference heterogeneity was significant (*p* < 0.001) across all attributes and levels except for 70% screening accuracy (*β* = −0.066, *p* > 0.05) and the 5‐year screening interval (*β* = 0.135, *p* > 0.05). Details are provided in Table 4.

**TABLE 4:** Analysis of preference heterogeneity.

Attributes and levels	Ref.	Recipients perspective	Providers perspective
		Coefficient (95% CI)	Coefficient (95% CI)
Bowel preparation			
Yes	No	1.378*** (1.153, 1.602)	1.053*** (0.830, 1.277)
Screening accuracy			
50%	90%	1.082*** (0.749, 1.415)	0.940*** (0.643, 1.236)
70%		1.290*** (0.988, 1.592)	−0.066 (−1.183, 1.052)
Screening interval			
Annually	Every 10 years	1.891*** (1.521, 2.261)	1.263*** (0.917, 1.608)
Every 2 years		1.473*** (1.128, 1.819)	1.071*** (0.726, 1.415)
Every 5 years		1.249*** (0.936, 1.561)	0.135 (−0.323, 0.594)
Reduction in CRC‐related mortality			
10%	90%	1.019*** (0.727, 1.311)	0.792*** (0.465, 1.119)
50%		−1.502*** (−1.807, −1.197)	1.052*** (0.756, 1.348)

*Note:* The sign of the estimated standard deviations is irrelevant: interpret them as being positive.

*p* < 0.05 is considered statistically significant (*p < 0.05; **p < 0.01; ***p < 0.001).

### 3.3 Analysis of Willingness to Pay

Estimates from the mixed logit model indicate that Recipients have the highest willingness to pay for shorter screening intervals. When the interval is shortened from 10 years to 1, 2, and 5 years, Recipients are willing to pay an additional ¥907.98, ¥1052.95, and ¥485.56, respectively. For a reduction from 10 to 2 years, Providers expect to charge an additional ¥1370.84. Additionally, reductions in screening accuracy and decreases in CRC‐related mortality reduction correspond to lower willingness to pay by Recipients and lower charges expected by Providers. Details are presented in Table 5.

**TABLE 5:** Willingness to pay estimates for the study population.

Attributes and levels	*β* (95% CI)	
	Recipients perspective	Providers perspective
Bowel preparation		
No (Ref.)		
Yes	140.8929* (19.141, 262.645)	1406.933*** (872.057, 1941.809)
Screening accuracy		
90% (Ref.)		
70%	−649.6199*** (−814.245, −484.995)	−1774.691*** (−2308.121, −1169.261)
50%	−900.877*** (−1070.940, −730.814)	−1827.201*** (−2445.460, −1208.942)
Screening interval		
Every 10 years (Ref.)		
Every 5 years	485.563*** (324.691, 646.436)	—
Every 2 years	1052.949*** (832.200, 1273.698)	1370.842*** (842.143, 1899.542)
Annually	907.976*** (687.246, 1128.706)	—
Reduction in CRC‐related mortality		
90% (Ref.)		
50%	−347.2249*** (−505.915, −188.535)	−1595.292*** (−2244.597, −945.987)
10%	—	−1341.161*** (−1960.563, −721.759)

*Note:* “—” denotes that the MWTP is not reported because the corresponding mean coefficient was not statistically significant.

*p* < 0.05 is considered statistically significant (*p < 0.05; **p < 0.01; ***p < 0.001).

### 3.4 Expected Participation Rate

**FIGURE 1:**
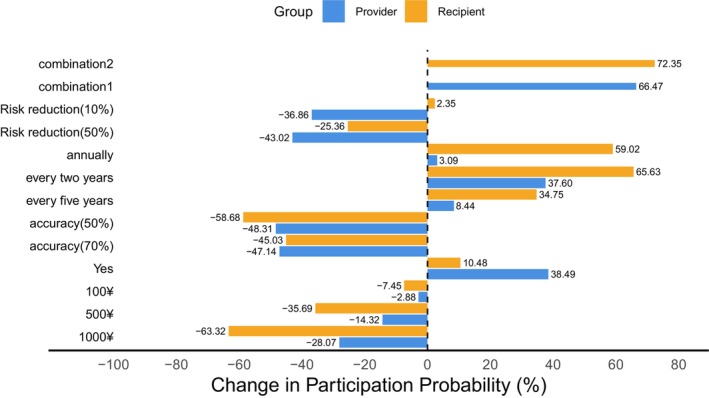
Expected participation rates for different attributes and levels from the recipients and providers perspectives. (1) Combinations 1 and 2 denote the changes in expected participation rates of the most preferred attribute combinations, from the Recipients and Providers perspectives respectively, relative to the reference combination. (2) Estimated uptake rates reflect conditional choice probabilities for a given alternative across two scenarios, excluding the opt‐out option.

The expected participation rate refers to the predicted probability of choice derived from the preference model, representing the potential acceptability and participation level of a given screening program within the target population. The study used baseline levels of zero screening costs, no bowel preparation, 90% screening accuracy, a 10‐year screening interval, and a 90% reduction in CRC‐related mortality. Changes in screening uptake under varying attribute levels are shown in Figure 1. When screening costs increase from zero to ¥1000, uptake rates among Recipients and Providers decrease by 63.32% and 28.07%, respectively. Shortening the screening interval from 10 to 2 years results in the largest increase in Recipients' uptake, by 65.63%, while Providers show the greatest increase, 38.49%, when bowel preparation is included. Under the combination of zero screening costs, bowel preparation, 90% screening accuracy, a 2‐year interval, and 90% mortality reduction (combination 1), Providers' uptake increases by 66.47%. For zero costs, bowel preparation, 90% accuracy, a 2‐year interval, and 10% mortality reduction (combination 2), Recipients' uptake rises by 72.35%.

### 3.5 Subgroup Analysis

Recipients in Weifang (*β* = −0.157, *p* > 0.05), those without a family history of cancer (*β* = 0.133, *p* > 0.05), and females (*β* = 0.110, *p* > 0.05) did not exhibit significant preferences for bowel preparation. Compared with a 90% reduction in CRC‐related mortality, recipients in Weifang showed a significant positive preference for a 10% reduction (*β* = 0.359, *p* < 0.05). In addition, providers in Jining did not demonstrate significant preferences for screening costs (*β* = −0.000, *p* > 0.05) (Tables S7–S9).

## 4 Discussion

The results indicated that both Recipients and Providers exhibited stronger preferences for CRC screening when the out‐of‐pocket or charged screening costs were lower, the screening interval was every 2 years, bowel preparation was included, test accuracy was higher, and mortality risk reduction was greater (ASC = −1.917, *p* < 0.001) (Table 3) [38]. Interestingly, Recipients did not exhibit significant aversion to bowel preparation, suggesting they may associate it with higher screening quality and professionalism. Additionally, Providers showed a strong preference for screening with bowel preparation (RIS = 35.4%), which may partly explain the lack of aversion among Recipients [39]. However, Recipients in Weifang did not exhibit a significant preference for bowel preparation. As a relatively developed region and a provincial model for CRC screening, Weifang initiated screening programs earlier, with more comprehensive policies, better resource accessibility, and higher levels of health literacy among its residents. Consequently, local Recipients may prioritize other attributes and regard bowel preparation as a secondary factor. In addition, the potential association between bowel preparation and complication risks may further contribute to the lack of significant preference [40].

Previous studies have shown that economic status affects CRC screening uptake, consistent with our findings [41, 42, 43]. We found that among all attributes, screening costs were the primary factor influencing Recipients' participation (RIS = 42.8%). Changes in attribute levels led to corresponding variations in their willingness to pay. Therefore, policymakers should give full consideration to Recipients' economic circumstances when designing screening strategies. In addition, screening interval also significantly influenced decisions for both Recipients (RIS = 24.3%) and Providers (RIS = 31.7%). As the gold standard for screening, colonoscopy is typically recommended at intervals of 5–10 years. However, in contrast to Recipients' preference for shorter screening intervals, Providers exhibited a stronger preference for biennial screening, although considerable heterogeneity was observed among individuals. This heterogeneity may stem from differing perceptions among Providers regarding screening methods [44], results [9], repeat screenings [45], resource constraints [46], and potential adverse effects on patients [47]. It is noteworthy that risk assessment plays a critical role in clinical decision‐making; yet Providers rarely consider risk factors beyond age [48], which is consistent with our findings. In our study, the reduction in CRC‐related mortality ranked only fourth in terms of RIS among Providers, and they even demonstrated a significant negative preference toward a 10% reduction in mortality risk. By contrast, Recipients exhibited no clear average effect for the same attribute (*p* > 0.05). This discrepancy may be attributed to information asymmetry between Providers and Recipients. Prior evidence suggests that nearly all Providers initially recommend colonoscopy for CRC screening, while FOBT is often proposed for those who decline colonoscopy [48]. Such practice may reinforce information asymmetry, limiting Recipients' ability to make informed decisions. Therefore, in the promotion of CRC screening, Providers should deliver comprehensive information to Recipients and adequately consider their preferences to facilitate shared decision‐making.

This study has several strengths. First, to our knowledge, this preference survey conducted in Shandong Province, China, is the first to examine the preferences from both supply and demand sides—namely, community residents (Recipients) and healthcare professionals (Providers)—in the context of CRC screening [49]. These findings are of global relevance for optimizing CRC screening strategies, particularly in developing countries with a high incidence and mortality of CRC. Second, our sample size far exceeded the minimum recommendation of 20 respondents per version in the DCE user guide, thereby enhancing the representativeness of the results and providing evidence that can be referenced in other countries or regions. Finally, given the limited availability of real‐world screening data, our use of stated preferences from both Recipients and Providers overcomes the constraints of scarce or incomplete records.

However, several limitations should also be acknowledged. First, our study primarily focused on the overall preference differences between Recipients and Providers, without fully addressing subgroup heterogeneity—particularly differences across economic levels among Recipients. This will be an important direction for future research. Second, we did not include an opt‐out option, which may have led to an overestimation of expected outcomes. Nonetheless, this approach enabled the model to focus more clearly on the relative effects of screening attributes, thereby improving estimation stability, aligning with policy simulation needs, and providing a more intuitive assessment of how different screening strategies influence participation.

In short, preferences for CRC screening attributes and levels differ between Recipients and Providers. These differences provide critical insights for the optimization of screening strategies. By accounting for both perspectives, our study offers practical implications for maximizing the effectiveness of CRC screening and improving adherence. Importantly, future research should further explore subgroup heterogeneity across different characteristics to promote personalized and precise screening strategies.

## 5 Conclusion

Screening costs, bowel preparation, screening accuracy, screening interval, and reduction in CRC‐related mortality all played significant roles in CRC screening decisions among both Recipients and Providers. Policymakers should carefully consider and balance preferences for these attributes, with particular attention to the economic status of Recipients. In addition, Providers should recognize the needs and preferences of Recipients and ensure that comprehensive information is communicated prior to screening to facilitate shared decision‐making.

## Author Contributions

**Weimin Guan:** conceptualization (lead), formal analysis (lead), investigation (lead), methodology (lead), writing – original draft (lead), writing – review and editing (lead). **Nan Zhang:** conceptualization (supporting), formal analysis (supporting), investigation (supporting), methodology (supporting), writing – original draft (supporting), writing – review and editing (supporting). **Zhengyang Lu:** methodology (supporting), writing – review and editing (supporting). **Mingjun Zhang:** methodology (supporting), writing – review and editing (supporting). **Yan Liu:** methodology (supporting), writing – review and editing (supporting). **Pengfei Li:** methodology (supporting), writing – review and editing (supporting). **Hang Yu:** investigation (supporting). **Boyu Liu:** investigation (supporting). **Wenxuan Yan:** investigation (supporting). **Guifeng Ma:** conceptualization (supporting), funding acquisition (supporting), methodology (supporting), writing – review and editing (supporting). **Youhua Lu:** conceptualization (supporting), funding acquisition (supporting), methodology (supporting), writing – review and editing (supporting).

## Ethics Statement

This study was approved by the Ethics Committee of the Affiliated Cancer Hospital of Shandong First Medical University (Approval No.: SDTHEC2024003167).

## Consent

Informed consent was obtained from all individual participants included in the study.

## Conflicts of Interest

The authors declare no conflicts of interest.

## Supporting information


**Data S1:** Supplementary Information.

## Data Availability

The data that support the findings of this study are available on request from the corresponding author. The data are not publicly available because of privacy and ethical restrictions.
