# Supplementary material for: Colorectal Cancer Screening Preferences of Recipients and Providers: A Dual‐Perspective Discrete Choice Experiment
Source: Cancer Med. 2025 Nov 2;14(21):e71341. doi: 10.1002/cam4.71341 (PMC12579823; doi:10.1002/cam4.71341)
Supplement: Supplementary file 1 — Data S1: Supplementary Information. [file CAM4-14-e71341-s001.docx]

**Supplementary Material**

1. **Supplementary Tables**

| **Table S1. The attributes and their levels initially identified in the literature review** | |
| --- | --- |
| **Primary attribute** | **Level** |
| Prepare | 4L laxative; 2L laxative +1L clear liquid; 1L Laxative +1L clear liquid; 0.3L laxative +2L clear liquid |
|  | None; Enema; Drink 2 to 4 liters of water and then fast |
|  | None; minimum; density |
|  | None; Laxative; Enema; Diet + laxatives |
|  | None; Special diet; Laxative |
|  | None; Diet; Enemas or laxatives |
| Location | Home; Hospital; Pathology Center |
| Risk of complications | None; small |
| Type | Colonoscopy; Home fecal test; Blood test |
| Frequency/Interval | Once a year; once every three years; once every ten years |
|  | Once a year; once every two years; once every five years |
|  | Once a year; once every five years; once every ten years |
|  | Once a year; once every two years; once every three years |
| True positive rate | 60%; 70%; 80%; 90%; 100% |
| Sensitivity | 40%; 70%; 90% |
| True negative rate | 70%; 80%; 90%; 100% |
| Specificity | 50%; 80%; 100% |
| Genetic susceptibility probability | 1%; 3%; 15% |
| The probability of having CRC | 15%; 70%; 99% |
|  | 25%; 50% |
| Test accuracy | 60%; 75%; 90% |
| True positive rate of adenoma (for doctors only) | 20%; 50%; 100% |
| CRC survival rate | 80%; 92%; 98% |
| Transportation demand | Never; Occasionally; Almost always |
| Pain | None; Slight |
| The risk of CRC-related deaths is reduced | 0%; 40%; 80% |
| Reduced risk of CRC | 8%→4%; 8%→2%; 8%→1% |
|  | 8%→6%; 8%→4%; 8%→2%; 8%→1% |
| Out-of-pocket expenses | $0; $93; $186 |
|  | $250; $500; $1,000 or $1,500 |
|  | $0; $75; $150 |
|  | $10; $50; $250; $500 |
|  | $30; $50; $80 |

**Dear expert:**

Hello! Thank you very much for accepting our consultation in your busy schedule. The Discrete Choice Experiment (DCE) is a research method originated from consumer behavior science. It is used to evaluate and analyze an individual's preferences when making choices among multiple options. We are conducting a study on the preferences of both supply and demand sides for colorectal cancer screening, aiming to analyze the key factors (i.e., attributes) influencing the participation rate of screening through DCEs, and based on this, formulate more scientific and reasonable screening strategies to increase the participation rate and coverage of screening and facilitate the early detection and control of colorectal cancer. To ensure the scientific and practical nature of the research design, we plan to conduct an in-depth interview with you. We are now seeking your valuable opinions and suggestions on the determination of attributes and levels. Your valuable opinions are of vital importance to our research. We will strictly adhere to academic ethics, ensure that all information is only used for academic research, and keep it strictly confidential. Thank you again for your support and help!

Based on your experience, please help us assess the rationality and feasibility of these attributes and their levels, and offer suggestions for improvement.

| **Table S2. Consultation list** | | | |
| --- | --- | --- | --- |
| **Attribute** | **Sorting column** | **Level** | **Suggestion** |
| Screening type |  | Fecal occult blood test; Colonoscopy; Fecal DNA and other new technologies |  |
| Screening preparation |  | None; Diet; Laxative |  |
| Screening sensitivity |  | 40%; 70%; 90% |  |
| Screening specificity |  | 50%; 80%; 100% |  |
| Screening location |  | Home; Hospital |  |
| Screening interval |  | Once a year; Once every two years; Once every five years |  |
| Out-of-pocket expenses |  | 0 yuan; 200 yuan; 500 yuan |  |
| Pain |  | None; Slight |  |
| Risk of complications |  | None; small |  |
| Reduction in CRC-related mortality |  | 0%; 40%； 80% |  |

| **Table S3. Expert Details** | |
| --- | --- |
| **Expert type** | **Number of experts** |
| Project leader of the cancer screening program | 2 |
| Director of the Endoscopy Department | 6 |
| Community evangelists | 1 |
| Policy management expert | 1 |
| DCE expert | 2 |
| Total | 12 |

**Material 1**

**Colorectal Cancer Screening Preference Survey**

**Questionnaire Instructions**

**Project Introduction**

Dear interviewee, hello! We are the Colorectal Cancer Screening Preference Research Team of Shandong Cancer Hospital. We are currently conducting a preference survey on colorectal cancer screening from the perspective of supply and demand. We sincerely invite you to participate in this research. Colorectal Cancer has brought a huge disease burden and economic burden to society and has become a major global public health problem. Early detection, early intervention and early treatment can effectively reduce the risk of death from colorectal cancer and increase the possibility of successful treatment and survival. Therefore, this survey aims to provide a practical basis for exploring the optimal colorectal cancer screening program suitable for China's national conditions by obtaining your preferences for colorectal cancer screening.

**Information collection and use**

Your choice to participate will mean that you accept a questionnaire survey. The self-made questionnaire will involve your personal basic information and other contents. Our questionnaire survey will take up approximately 5 minutes of your time. All the survey information collected during the research process will be strictly confidential. There are no right or wrong answers or options. Please fill in your actual situation with confidence and do not leave any blanks.

**Signature**

I have clearly read and understood the above information, and all my questions have been answered satisfactorily. I am also satisfied with the explanations given by the investigator. I understand: My participation is voluntary, and I can interrupt my participation at any time. Signing this informed consent form does not mean waiving any of my legal rights. I will participate in this research.

1.　 If you have read and understood the above content and are willing to participate in our survey, please fill in your name here. 【 】

2.　 Your gender

　　□ Male　□Female

3.　 How old are you? (In years) 【 】

4.　 Your educational level

□ Illiterate or poorly literate　□ Primary school　□ Junior high school　□ High school or vocational high school or technical secondary

school　□ Junior college　□ Undergraduate　□Graduate student

5.　 Your marital status

□ Married and living with a spouse　□ Married, but temporarily not living with the spouse due to work or other reasons　□ Separation (no

longer living together as spouses)　□Divorce　□ Widowed　□Never married

6.　 Has anyone among your blood relatives (parents, grandparents, maternal grandparents, siblings, uncles and aunts, Cousins, and Cousins) ever

had cancer?

□ Yes　□No

7.　 Your location

□ Weifang　□ Tai 'an　□Jining

8.　 Your professional title (medical staff)

□ None　□Junior professional title　□ Intermediate professional title　□Associate senior title or above

Based on the relevant information listed, please comprehensively consider the five attributes and select the option you prefer from the two options of each screening plan below. Then, further decide whether you are willing to participate in the screening according to the selected plan in real life. There are a total of 7 sets of plans. Among them, Option 1 and Option 2 are two different colorectal cancer screenings, which differ in different attributes (i.e., Bowel preparation, screening accuracy, screening interval, screening costs, and reduction in CRC-related mortality).

**Attribute explanation**

1. Bowel preparation: The process of cleansing the bowel of faeces and residual contents through a series of preparatory measures prior to colorectal cancer screening.

Level: Yes（This includes controlling diet and taking intestinal cleansers, such as laxatives.）; No

2. Screening accuracy: The ability of screening to detect colorectal cancer and its precancerous lesions.

Level: 50%; 70%; 90%

3. Screening interval: The interval between two consecutive screening tests.

Level: Annually; Every two years; Every five years; Every ten years

4. Screening costs: The cost charged by healthcare providers or paid out-of-pocket by individuals during the colorectal cancer screening process.

Level: 0￥; 100￥; 500￥; 1000￥

5. Reduction in CRC-related mortality: Colorectal cancer screening facilitates the early detection of colorectal cancer or precancerous lesions, enabling timely and effective interventions that can reduce the risk of mortality associated with colorectal cancer.

Level: 10%; 50%; 90%

**Plan 1**

| **Attributes** | **Option 1** | **Option 2** |
| --- | --- | --- |
| Bowel preparation | No | Yes |
| Screening accuracy | 70% | 90% |
| Screening interval | Every two years | Annually |
| Screening costs | 0￥ | 500￥ |
| Reduction in CRC-related mortality | 10% | 50% |
| 1. Which one would you prefer to choose?　　□ Option 1　　□ Option 2 | | |
| 2. In real life, would you be willing to participate in the screening according to the selected plan?　　□Yes　　□No | | |

**Plan 2**

| **Attributes** | **Option 1** | **Option 2** |
| --- | --- | --- |
| Bowel preparation | Yes | No |
| Screening accuracy | 90% | 70% |
| Screening interval | Every two years | Every five years |
| Screening costs | 500￥ | 1000￥ |
| Reduction in CRC-related mortality | 10% | 50% |
| 1. Which one would you prefer to choose?　　□ Option 1　　□ Option 2 | | |
| 2. In real life, would you be willing to participate in the screening according to the selected plan?　　□Yes　　□No | | |

**Plan 3**

| **Attributes** | **Option 1** | **Option 2** |
| --- | --- | --- |
| Bowel preparation | Yes | No |
| Screening accuracy | 90% | 50% |
| Screening interval | Every five years | Annually |
| Screening costs | 100￥ | 500￥ |
| Reduction in CRC-related mortality | 90% | 50% |
| 1. Which one would you prefer to choose?　　□ Option 1　　□ Option 2 | | |
| 2. In real life, would you be willing to participate in the screening according to the selected plan?　　□Yes　　□No | | |

**Plan 4**

| **Attributes** | **Option 1** | **Option 2** |
| --- | --- | --- |
| Bowel preparation | Yes | No |
| Screening accuracy | 50% | 70% |
| Screening interval | Every two years | Every five years |
| Screening costs | 1000￥ | 500￥ |
| Reduction in CRC-related mortality | 50% | 10% |
| 1. Which one would you prefer to choose?　　□ Option 1　　□ Option 2 | | |
| 2. In real life, would you be willing to participate in the screening according to the selected plan?　　□Yes　　□No | | |

**Plan 5**

| **Attributes** | **Option 1** | **Option 2** |
| --- | --- | --- |
| Bowel preparation | Yes | No |
| Screening accuracy | 70% | 90% |
| Screening interval | Every ten years | Every five years |
| Screening costs | 100￥ | 1000￥ |
| Reduction in CRC-related mortality | 90% | 10% |
| 1. Which one would you prefer to choose?　　□ Option 1　　□ Option 2 | | |
| 2. In real life, would you be willing to participate in the screening according to the selected plan?　　□Yes　　□No | | |

**Plan 6**

| **Attributes** | **Option 1** | **Option 2** |
| --- | --- | --- |
| Bowel preparation | No | Yes |
| Screening accuracy | 50% | 70% |
| Screening interval | Every ten years | Annually |
| Screening costs | 100￥ | 0￥ |
| Reduction in CRC-related mortality | 10% | 90% |
| 1. Which one would you prefer to choose?　　□ Option 1　　□ Option 2 | | |
| 2. In real life, would you be willing to participate in the screening according to the selected plan?　　□Yes　　□No | | |

**Plan 7**

| **Attributes** | **Option 1** | **Option 2** |
| --- | --- | --- |
| Bowel preparation | Yes | No |
| Screening accuracy | 90% | 70% |
| Screening interval | Every two years | Every five years |
| Screening costs | 500￥ | 1000￥ |
| Reduction in CRC-related mortality | 10% | 50% |
| 1. Which one would you prefer to choose?　　□ Option 1　　□ Option 2 | | |
| 2. In real life, would you be willing to participate in the screening according to the selected plan?　　□Yes　　□No | | |

**Do you understand making a choice among different screening programs?**

□Fully understand

□Relatively understand

□General

□I don't quite understand

□Don't understand at all

| **Table S4. Model Fit Comparison of Mixed Logit Models at Different nrep Values (Recipient Perspective)** | | | | | | | | |
| --- | --- | --- | --- | --- | --- | --- | --- | --- |
| **Indicator** | **nrep = 50** | | **nrep = 200** | | **nrep = 500** | | **nrep = 1000** | |
|  | **Model 1** | | **Model 1** | | **Model 1** | | **Model 1** | |
|  | ***Mean*** | ***SD*** | ***Mean*** | ***SD*** | ***Mean*** | ***SD*** | ***Mean*** | ***SD*** |
| Screening costs | -0.001***  (-0.001, -0.001) |  | -0.001***  (-0.002, -0.001) |  | -0.002***  (-0.002, -0.001) |  | -0.002***  (-0.002, -0.001) |  |
| ASC | -1.636***  (-1.850, -1.422) |  | -1.853***  (-2.111, -1.596) |  | -1.992***  (-2.274, -1.711) |  | -2.093***  (-2.399, -1.787) |  |
| Bowel preparation |  |  |  |  |  |  |  |  |
| No (*ref.*) |  |  |  |  |  |  |  |  |
| Yes | 0.172*  (0.032, 0.311) | 1.104***  (0.923, 1.286) | 0.235**  (0.067, 0.403) | 1.415***  (1.179, 1.650) | 0.260**  (0.080, 0.439) | 1.466***  (1.216, 1.716) | 0.257**  (0.068, 0.446) | 1.564***  (1.288, 1.840) |
| Screening accuracy |  |  |  |  |  |  |  |  |
| 90% (*ref.*) |  |  |  |  |  |  |  |  |
| 70% | -0.834***  (-1.012, -0.656) | 0.988***  (0.731, 1.246) | -0.981***  (-1.202, -0.759) | 1.369***  (1.062, 1.675) | -1.054***  (-1.296, -0.812) | 1.511***  (1.175, 1.847) | -1.085***  (-1.336, -0.835) | 1.544***  (1.191, 1.897) |
| 50% | -1.135***  (-1.317, -0.952) | 0.606**  (0.236, 0.977) | -1.341***  (-1.578, -1.105) | 1.076***  (0.771, 1.380) | -1.423***  (-1.675, -1.170) | 1.040***  (0.689, 1.391) | -1.515***  (-1.794, -1.236) | 1.173***  (0.798, 1.547) |
| Screening interval |  |  |  |  |  |  |  |  |
| Every ten years (*ref.*) |  |  |  |  |  |  |  |  |
| Every five years | 0.609***  (0.422, 0.796) | 0.898***  (0.597, 1.200) | 0.671***  (0.450, 0.893) | 1.242***  (0.922, 1.562) | 0.740***  (0.502, 0.977) | 1.386***  (1.044, 1.727) | 0.781***  (0.528, 1.033) | 1.483***  (1.123, 1.842) |
| Every two years | 1.336***  (1.127, 1.545) | 1.035***  (0.729, 1.341) | 1.529***  (1.274, 1.784) | 1.365***  (1.007, 1.722) | 1.628***  (1.349, 1.908) | 1.527***  (1.148, 1.906) | 1.711***  (1.406, 2.015) | 1.683***  (1.269, 2.096) |
| Annually | 1.106***  (0.896, 1.316) | 1.448***  (1.148, 1.747) | 1.325***  (1.062, 1.587) | 1.841***  (1.491, 2.190) | 1.393***  (1.104, 1.681) | 2.048***  (1.652, 2.445) | 1.454***  (1.147, 1.761) | 2.156***  (1.738, 2.574) |
| Reduction in CRC-related mortality |  |  |  |  |  |  |  |  |
| 90% (*ref.*) |  |  |  |  |  |  |  |  |
| 50% | -0.449***  (-0.628, -0.271) | 1.128***  (0.881, 1.375) | -0.517***  (-0.734, -0.299) | 1.502***  (1.197, 1.807) | -0.604***  (-0.839, -0.368) | 1.586***  (1.267, 1.905) | -0.606***  (-0.853, -0.359) | 1.694***  (1.352, 2.035) |
| 10% | -0.033  (-0.209, 0.143) | 0.718***  (0.466, 0.969) | -0.014  (-0.221, 0.193) | 0.960***  (0.652, 1.268) | 0.016  (-0.206, 0.237) | 0.983***  (0.664, 1.302) | 0.027  (-0.202, 0.256) | 1.077***  (0.756, 1.398) |
| Sample size | 594 | | 594 | | 594 | | 594 | |
| Number of observations | 10692 | | 10692 | | 10692 | | 10692 | |
| LL | -3115.5889 | | -3088.5325 | | -3076.749 | | -3070.2772 | |
| AIC | 6267.1777 | | 6213.065 | | 6189.498 | | 6176.5543 | |
| BIC | 6346.1416 | | 6292.0289 | | 6268.4618 | | 6255.5182 | |
| **Note:** (1) Model 1 includes the entire sample, encompassing respondents who did not pass the consistency test; Model 2 excludes respondents who failed the consistency test. LL: Log-likelihood; AIC: Akaike Information Criterion; BIC: Bayesian Information Criterion. (2) *p* < 0.05 is considered statistically significant. (* *p* < 0.05; ** *p* < 0.01; *** *p* < 0.001) (3) The sign of the estimated standard deviations is irrelevant: interpret them as being positive. | | | | | | | | |

| **Table S4 Continued. Model Fit Comparison of Mixed Logit Models at Different nrep Values (Recipient Perspective)** | | | | | | | | |
| --- | --- | --- | --- | --- | --- | --- | --- | --- |
| **Indicator** | **nrep = 1200** | | **nrep = 1500** | | **nrep = 1800** | | **nrep = 2000** | |
|  | **Model 1** | | **Model 1** | | **Model 1** | | **Model 1** | |
|  | ***Mean*** | ***SD*** | ***Mean*** | ***SD*** | ***Mean*** | ***SD*** | ***Mean*** | ***SD*** |
| Screening costs | -0.002***  (-0.002, -0.001) |  | -0.002***  (-0.002, -0.001) |  | -0.002***  (-0.002, -0.001) |  | -0.002***  (-0.002, -0.001) |  |
| ASC | -2.075***  (-2.376, -1.775) |  | -2.142***  (-2.460, -1.825) |  | -2.149***  (-2.469, -1.830) |  | -2.112***  (-2.422, -1.803) |  |
| Bowel preparation |  |  |  |  |  |  |  |  |
| No (*ref.*) |  |  |  |  |  |  |  |  |
| Yes | 0.254**  (0.065, 0.442) | 1.553***  (1.280, 1.826) | 0.279**  (0.084, 0.474) | 1.603***  (1.314, 1.891) | 0.274**  (0.078, 0.469) | 1.601***  (1.321, 1.881) | 0.284**  (0.089, 0.479) | 1.585***  (1.306, 1.864) |
| Screening accuracy |  |  |  |  |  |  |  |  |
| 90% (*ref.*) |  |  |  |  |  |  |  |  |
| 70% | -1.113***  (-1.369, -0.858) | 1.482***  (1.138, 1.826) | -1.124***  (-1.388, -0.861) | 0.567***  (1.204, 1.930) | -1.133***  (-1.395, -0.870) | 1.595***  (1.235, 1.954) | -1.134***  (-1.398, -0.869) | 1.589***  (1.222, 1.957) |
| 50% | -1.488***  (-1.761, -1.215) | 1.143***  (0.791, 1.495) | -1.553***  (-1.843, -1.263) | 1.224***  (0.865, 1.584) | -1.566***  (-1.859, -1.273) | 1.210***  (0.849, 1.572) | -1.536***  (-1.820, -1.252) | 1.156***  (0.793, 1.519) |
| Screening interval |  |  |  |  |  |  |  |  |
| Every ten years (*ref.*) |  |  |  |  |  |  |  |  |
| Every five years | 0.752***  (0.504, 1.000) | 1.467***  (1.118, 1.816) | 0.784***  (0.527, 1.041) | 1.535***  (1.154, 1.916) | 0.776***  (0.521, 1.031) | 1.502***  (1.138, 1.865) | 0.769***  (0.517, 1.022) | 1.484***  (1.110, 1.859) |
| Every two years | 1.702***  (1.401, 2.002) | 1.678***  (1.263, 2.093) | 1.750***  (1.432, 2.069) | 1.774***  (1.354, 2.194) | 1.783***  (1.459, 2.107) | 1.794***  (1.370, 2.218) | 1.759***  (1.438, 2.081) | 1.740***  (1.317, 2.162) |
| Annually | 1.481***  (1.172, 1.791) | 2.164***  (1.748, 2.580) | 1.524***  (1.199, 1.849) | 2.241***  (1.802, 2.680) | 1.503***  (1.184, 1.821) | 2.236***  (1.799, 2.672) | 1.491***  (1.173, 1.809) | 2.256***  (1.809, 2.703) |
| Reduction in CRC-related mortality |  |  |  |  |  |  |  |  |
| 90% (*ref.*) |  |  |  |  |  |  |  |  |
| 50% | -0.604***  (-0.853, -0.355) | 1.720***  (1.368, 2.071) | -0.639***  (-0.901, -0.378) | 1.837***  (1.459, 2.215) | -0.608***  (-0.864, -0.352) | 1.816***  (1.437, 2.194) | -0.633***  (-0.888, -0.378) | 1.747***  (1.388, 2.106) |
| 10% | 0.016  (-0.213, 0.245) | 1.098***  (0.772, 1.425) | 0.010  (-0.225, 0.245) | 1.106***  (0.777, 1.435) | 0.018  (-0.218, 0.253) | 1.158***  (0.824, 1.493) | -0.003  (-0.237, 0.231) | 1.158***  (0.816, 1.499) |
| Sample size | 594 | | 594 | | 594 | | 594 | |
| Number of observations | 10692 | | 10692 | | 10692 | | 10692 | |
| LL | -3068.6433 | | -3066.5592 | | -3065.8179 | | -3068.4898 | |
| AIC | 6173.2866 | | 6169.1183 | | 6167.6357 | | 4172.9795 | |
| BIC | 6252.2504 | | 6248.0822 | | 6246.5995 | | 6251.9433 | |
| **Note:** (1) Model 1 includes the entire sample, encompassing respondents who did not pass the consistency test; Model 2 excludes respondents who failed the consistency test. LL: Log-likelihood; AIC: Akaike Information Criterion; BIC: Bayesian Information Criterion. (2) *p* < 0.05 is considered statistically significant. (* *p* < 0.05; ** *p* < 0.01; *** *p* < 0.001) (3) The sign of the estimated standard deviations is irrelevant: interpret them as being positive. | | | | | | | | |

| **Table S4 Continued. Model Fit Comparison of Mixed Logit Models at Different nrep Values (Recipient Perspective)** | | | | | | | | |
| --- | --- | --- | --- | --- | --- | --- | --- | --- |
| **Indicator** | **nrep = 50** | | **nrep = 200** | | **nrep = 500** | | **nrep = 1000** | |
|  | **Model 2** | | **Model 2** | | **Model 2** | | **Model 2** | |
|  | ***Mean*** | ***SD*** | ***Mean*** | ***SD*** | ***Mean*** | ***SD*** | ***Mean*** | ***SD*** |
| Screening costs | -0.001***  (-0.001, -0.001) |  | -0.001***  (-0.002, -0.001) |  | -0.002***  (-0.002, -0.001) |  | - |  |
| ASC | -1.654***  (-1.874, -1.434) |  | -1.917***  (-2.183, -1.652) |  | -1.983***  (-2.269, -1.697) |  | - |  |
| Bowel preparation |  |  |  |  |  |  |  |  |
| No (*ref.*) |  |  |  |  |  |  |  |  |
| Yes | 0.208**  (0.064, 0.351) | 1.169***  (0.976, 1.362) | 0.210*  (0.036, 0.385) | 1.378***  (1.153, 1.602) | 0.237*  (0.051, 0.423) | 1.476***  (1.212, 1.741) | - | - |
| Screening accuracy |  |  |  |  |  |  |  |  |
| 90% (*ref.*) |  |  |  |  |  |  |  |  |
| 70% | -0.809***  (-0.987, -0.632) | 0.914***  (0.647, 1.180) | -0.970***  (-1.191, -0.750) | 1.290***  (0.988, 1.592) | -1.031***  (-1.275, -0.788) | 1.422***  (1.091, 1.753) | - | - |
| 50% | -1.134***  (-1.328, -0.939) | 0.849***  (0.578, 1.120) | -1.345***  (-1.585, -1.106) | 1.082***  (0.749, 1.415) | -1.433***  (-1.699, -1.166) | 1.169***  (0.825, 1.513) | - | - |
| Screening interval |  |  |  |  |  |  |  |  |
| Every ten years (*ref.*) |  |  |  |  |  |  |  |  |
| Every five years | 0.615***  (0.413, 0.816) | 1.113***  (0.828, 1.399) | 0.725***  (0.496, 0.955) | 1.249***  (0.936, 1.561) | 0.747***  (0.504, 0.991) | 1.388***  (1.039, 1.737) | - | - |
| Every two years | 1.387***  (1.172, 1.602) | 1.021***  (0.697, 1.345) | 1.573***  (1.312, 1.833) | 1.473***  (1.128, 1.819) | 1.696***  (1.401, 1.991) | 1.524***  (1.137, 1.910) | - | - |
| Annually | 1.182***  (0.959, 1.404) | 1.435***  (1.126, 1.744) | 1.356***  (1.088, 1.624) | 1.891***  (1.521, 2.261) | 1.431***  (1.135, 1.726) | 2.011***  (1.613, 2.409) | - | - |
| Reduction in CRC-related mortality |  |  |  |  |  |  |  |  |
| 90% (*ref.*) |  |  |  |  |  |  |  |  |
| 50% | -0.422***  (-0.610, -0.234) | 1.205***  (0.948, 1.462) | -0.519***  (-0.742, -0.296) | -1.502***  (-1.807, -1.197) | -0.578***  (-0.820, -0.336) | 1.624***  (1.282, 1.967) | - | - |
| 10% | -0.019  (-0.199, 0.161) | -0.497*  (-0.944, -0.049) | 0.047  (-0.170, 0.264) | 1.019***  (0.727, 1.311) | 0.028  (-0.197, 0.254) | 1.049***  (0.712, 1.386) | - | - |
| Sample size | 570 | | 570 | | 570 | | - | |
| Number of observations | 10260 | | 10260 | | 10260 | | - | |
| LL | -2977.3957 | | -2940.3346 | | -2942.0408 | | - | |
| AIC | 5990.7915 | | 5916.6692 | | 5920.0815 | | - | |
| BIC | 6069.013 | | 5994.8906 | | 5998.303 | | - | |
| **Note:** (1) Model 1 includes the entire sample, encompassing respondents who did not pass the consistency test; Model 2 excludes respondents who failed the consistency test. LL: Log-likelihood; AIC: Akaike Information Criterion; BIC: Bayesian Information Criterion. (2) *p* < 0.05 is considered statistically significant. (* *p* < 0.05; ** *p* < 0.01; *** *p* < 0.001) (3) The sign of the estimated standard deviations is irrelevant: interpret them as being positive. | | | | | | | | |

| **Table S5. Model Fit Comparison of Mixed Logit Models at Different nrep Values (Provider Perspective)** | | | | | | | | |
| --- | --- | --- | --- | --- | --- | --- | --- | --- |
| **Indicator** | **nrep = 50** | | **nrep = 200** | | **nrep = 500** | | **nrep = 1000** | |
|  | **Model 1** | | **Model 1** | | **Model 1** | | **Model 1** | |
|  | ***Mean*** | ***SD*** | ***Mean*** | ***SD*** | ***Mean*** | ***SD*** | ***Mean*** | ***SD*** |
| Screening costs | -0.000***  (-0.001, -0.000) |  | -0.001***  (-0.001, -0.000) |  | -0.001***  (-0.001, -0.000) |  | -0.001***  (-0.001, -0.000) |  |
| ASC | -3.128***  (-3.378, -2.879) |  | -3.190***  (-3.452, -2.927) |  | -3.304***  (-3.587, -3.021) |  | -3.309***  (-3.599, -3.019) |  |
| Bowel preparation |  |  |  |  |  |  |  |  |
| No (*ref.*) |  |  |  |  |  |  |  |  |
| Yes | 0.692***  (0.556, 0.829) | 0.872***  (0.677, 1.067) | 0.707***  (0.563, 0.852) | 0.920***  (0.718, 1.122) | 0.752***  (0.598, 0.905) | 1.016***  (0.807, 1.224) | 0.762***  (0.602, 0.922) | 1.026***  (0.806, 1.246) |
| Screening accuracy |  |  |  |  |  |  |  |  |
| 90% (*ref.*) |  |  |  |  |  |  |  |  |
| 70% | -0.870***  (-1.023, -0.717) | -0.153  (-0.498, 0.192) | -0.914***  (-1.074, -0.754) | 0.054  (-0.374, 0.481) | -0.972***  (-1.141, -0.804) | 0.052  (-0.783, 0.887) | -0.979***  (-1.152, -0.805) | 0.141  (-0.619, 0.901) |
| 50% | -0.960***  (-1.139, -0.780) | 0.663***  (0.374, 0.951) | -0.981***  (-1.167, -0.795) | 0.761***  (0.472, 1.049) | -1.043***  (-1.241, -0.844) | 0.913***  (0.635, 1.190) | -1.046***  (-1.248, -0.844) | 0.904***  (0.614, 1.195) |
| Screening interval |  |  |  |  |  |  |  |  |
| Every ten years (*ref.*) |  |  |  |  |  |  |  |  |
| Every five years | 0.157  (-0.014, 0.328) | 0.092  (-0.238, 0.421) | 0.162  (-0.014, 0.337) | 0.089  (-0.306, 0.484) | 0.192*  (0.007, 0.377) | -0.051  (-0.472, 0.370) | 0.189*  (0.004, 0.375) | 0.025  (-0.418, 0.467) |
| Every two years | 0.670***  (0.490, 0.850) | 0.736***  (0.407, 1.066) | 0.718***  (0.524, 0.912) | -0.916***  (-1.241, -0.591) | 0.760***  (0.554, 0.966) | 1.050***  (0.723, 1.378) | 0.770***  (0.558, 0.981) | 1.084***  (0.753, 1.415) |
| Annually | 0.044  (-0.143, 0.232) | 1.077***  (0.746, 1.408) | 0.061  (-0.133, 0.254) | 1.111***  (0.788, 1.433) | 0.066  (-0.137, 0.270) | 1.220***  (0.889, 1.551) | 0.071  (-0.134, 0.277) | 1.244***  (0.896, 1.592) |
| Reduction in CRC-related mortality |  |  |  |  |  |  |  |  |
| 90% (*ref.*) |  |  |  |  |  |  |  |  |
| 50% | -0.777***  (-0.942, -0.613) | 0.768***  (0.483, 1.054) | -0.829***  (-1.007, -0.652) | 0.859***  (0.576, 1.141) | -0.868***  (-1.057, -0.679) | 1.015***  (0.737, 1.293) | -0.868***  (-1.060, -0.676) | 1.005***  (0.722, 1.288) |
| 10% | -0.697***  (-0.868, -0.526) | 0.396  (-0.063, 0.856) | -0.714***  (-0.894, -0.535) | -0.597**  (-0.939, -0.256) | -0.734***  (-0.925, -0.543) | 0.696***  (0.389, 1.002) | -0.742***  (-0.936, -0.547) | 0.706***  (0.389, 1.024) |
| Sample size | 553 | | 553 | | 553 | | 553 | |
| Number of observations | 9954 | | 9954 | | 9954 | | 9954 | |
| LL | -2418.7104 | | -2415.4281 | | -2407.9893 | | -2407.697 | |
| AIC | 4873.4207 | | 4866.8563 | | 4851.9786 | | 4851.394 | |
| BIC | 4951.0972 | | 4944.5327 | | 4929.655 | | 4929.0705 | |
| **Note:** (1) Model 1 includes the entire sample, encompassing respondents who did not pass the consistency test; Model 2 excludes respondents who failed the consistency test. LL: Log-likelihood; AIC: Akaike Information Criterion; BIC: Bayesian Information Criterion. (2) *p* < 0.05 is considered statistically significant. (* *p* < 0.05; ** *p* < 0.01; *** *p* < 0.001) (3) The sign of the estimated standard deviations is irrelevant: interpret them as being positive. | | | | | | | | |

| **Table S5 Continued. Model Fit Comparison of Mixed Logit Models at Different nrep Values (Provider Perspective)** | | | | | | | | |
| --- | --- | --- | --- | --- | --- | --- | --- | --- |
| **Indicator** | **nrep = 1200** | | **nrep = 1500** | | **nrep = 1800** | | **nrep = 2000** | |
|  | **Model 1** | | **Model 1** | | **Model 1** | | **Model 1** | |
|  | ***Mean*** | ***SD*** | ***Mean*** | ***SD*** | ***Mean*** | ***SD*** | ***Mean*** | ***SD*** |
| Screening costs | -0.001***  (-0.001, -0.000) |  | - |  | - |  | - |  |
| ASC | -3.306  (-3.592, -3.019) |  | - |  | - |  | - |  |
| Bowel preparation |  |  |  |  |  |  |  |  |
| No (*ref.*) |  |  |  |  |  |  |  |  |
| Yes | 0.762***  (0.604, 0.920) | 1.025***  (0.810, 1.240) | - | - | - | - | - | - |
| Screening accuracy |  |  |  |  |  |  |  |  |
| 90% (*ref.*) |  |  |  |  |  |  |  |  |
| 70% | -0.977***  (-1.148, -0.809) | 0.094  (-0.566, 0.754) | - | - | - | - | - | - |
| 50% | -1.050***  (-1.252, -0.849) | 0.905***  (0.619, 1.191) | - | - | - | - | - | - |
| Screening interval |  |  |  |  |  |  |  |  |
| Every ten years (*ref.*) |  |  |  |  |  |  |  |  |
| Every five years | 0.189*  (0.004, 0.375) | 0.001  (-0.464, 0.466) | - | - | - | - | - | - |
| Every two years | 0.768***  (0.558, 0.978) | 1.076***  (0.747, 1.405) | - | - | - | - | - | - |
| Annually | 0.066  (-0.139, 0.270) | 1.224***  (0.888, 1.560) | - | - | - | - | - | - |
| Reduction in CRC-related mortality |  |  |  |  |  |  |  |  |
| 90% (*ref.*) |  |  |  |  |  |  |  |  |
| 50% | -0.867***  (-1.058, -0.676) | 1.014***  (0.734, 1.294) | - | - | - | - | - | - |
| 10% | -0.743***  (-0.936, -0.550) | 0.699***  (0.372, 1.026) | - | - | - | - | - | - |
| Sample size | 553 | | - | | - | | - | |
| Number of observations | 9954 | | - | | - | | - | |
| LL | -2408.5698 | | - | | - | | - | |
| AIC | 4853.1395 | | - | | - | | - | |
| BIC | 4930.816 | | - | | - | | - | |
| **Note:** (1) Model 1 includes the entire sample, encompassing respondents who did not pass the consistency test; Model 2 excludes respondents who failed the consistency test. LL: Log-likelihood; AIC: Akaike Information Criterion; BIC: Bayesian Information Criterion. (2) *p* < 0.05 is considered statistically significant. (* *p* < 0.05; ** *p* < 0.01; *** *p* < 0.001) (3) The sign of the estimated standard deviations is irrelevant: interpret them as being positive. | | | | | | | | |

| **Table S5 Continued. Model Fit Comparison of Mixed Logit Models at Different nrep Values (Provider Perspective)** | | | | | | | | |
| --- | --- | --- | --- | --- | --- | --- | --- | --- |
| **Indicator** | **nrep = 50** | | **nrep = 200** | | **nrep = 500** | | **nrep = 1000** | |
|  | **Model 2** | | **Model 2** | | **Model 2** | | **Model 2** | |
|  | ***Mean*** | ***SD*** | ***Mean*** | ***SD*** | ***Mean*** | ***SD*** | ***Mean*** | ***SD*** |
| Screening costs | -0.001***  （-0.001, -0.000） |  | -0.001***  （-0.001, -0.000） |  | -0.001***  (-0.001, -0.000) |  | -0.001***  (-0.001, -0.000) |  |
| ASC | -3.250***  （-3.514, -2.986） |  | -3.338***  （-3.620, -3.057） |  | -3.450***  (-3.756, -3.145) |  | -3.459***  (-3.774, -3.143) |  |
| Bowel preparation |  |  |  |  |  |  |  |  |
| No (ref.) |  |  |  |  |  |  |  |  |
| Yes | 0.683***  （0.546, 0.819） | 0.849***  （0.654, 1.043） | 0.743***  （0.591, 0.895） | 0.954***  （0.741, 1.168） | 0.812***  (0.641, 0.982) | 1.053***  (0.830, 1.277) | 0.803***  (0.634, 0.973) | 1.064***  (0.828, 1.301) |
| Screening accuracy |  |  |  |  |  |  |  |  |
| 90% (ref.) |  |  |  |  |  |  |  |  |
| 70% | -0.895***  （-1.053, -0.737） | 0.108  （-0.178, 0.395） | -0.952***  （-1.119, -0.785） | -0.014  （-0.487, 0.459） | -1.024***  (-1.203, -0.844) | -0.066  (-1.183, 1.052) | -1.023***  (-1.209, -0.837) | 0.120  (-0.531, 0.770) |
| 50% | -0.923***  （-1.098, -0.748） | 0.670***  （0.386, 0.955） | -1.004***  （-1.202, -0.807） | 0.843***  （0.549, 1.138） | -1.054***  (-1.262, -0.847) | 0.940***  (0.643, 1.236) | -1.069***  (-1.285, -0.853) | 0.953***  (0.641, 1.264) |
| Screening interval |  |  |  |  |  |  |  |  |
| Every ten years (ref.) |  |  |  |  |  |  |  |  |
| Every five years | 0.137  （-0.037, 0.311） | 0.019  （-0.334, 0.372） | 0.154  （-0.030, 0.338） | 0.008  (-0.382, 0.399) | 0.169  (-0.024, 0.362) | 0.135  (-0.323, 0.594) | 0.170  (-0.022, 0.363) | 0.098  (-0.391, 0.587) |
| Every two years | 0.699***  （0.512, 0.886） | 0.764***  （0.428, 1.100） | 0.750***  （0.548, 0.952） | 0.904***  (0.566, 1.242) | 0.791***  (0.574, 1.007) | 1.071***  (0.726, 1.415) | 0.795***  (0.574, 1.017) | 1.064***  (0.705, 1.424) |
| Annually | 0.042  （-0.143, 0.226） | 0.964***  （0.621, 1.306） | 0.041  （-0.159, 0.241） | 1.096***  （0.756, 1.435） | 0.062  (-0.153, 0.276) | 1.263***  (0.917, 1.608) | 0.050  (-0.163, 0.263) | 1.256***  (0.903, 1.609) |
| Reduction in CRC-related mortality |  |  |  |  |  |  |  |  |
| 90% (ref.) |  |  |  |  |  |  |  |  |
| 50% | -0.805***  （-0.975, -0.635） | -0.770***  （-1.051, -0.489） | -0.835***  （-1.019, -0.651） | 0.938***  (0.646, 1.229) | -0.920***  (-1.124, -0.716) | 1.052***  (0.756, 1.348) | -0.901***  (-1.106, -0.696) | 1.061***  (0.754, 1.369) |
| 10% | -0.685***  （-0.860, -0.511） | 0.549***  （0.250, 0.849） | -0.712***  （-0.902, -0.523） | 0.697***  (0.380, 1.015) | -0.774***  (-0.980, -0.567) | 0.792***  (0.465, 1.119) | -0.761***  (-0.964, -0.557) | 0.757***  (0.433, 1.082) |
| Sample size | 532 | | 532 | | 532 | | 532 | |
| Number of observations | 9576 | | 9576 | | 9576 | | 9576 | |
| LL | -2284.2273 | | -2277.6075 | | -2270.9724 | | -2272.4811 | |
| AIC | 4604.4545 | | 4591.215 | | 4577.9449 | | 4580.9621 | |
| BIC | 4681.4341 | | 4668.1946 | | 4654.9245 | | 4657.9417 | |
| Note: (1) Model 1 includes the entire sample, encompassing respondents who did not pass the consistency test; Model 2 excludes respondents who failed the consistency test. LL: Log-likelihood; AIC: Akaike Information Criterion; BIC: Bayesian Information Criterion. (2) *p* < 0.05 is considered statistically significant. (* *p* < 0.05; ** *p* < 0.01; *** *p* < 0.001) (3) The sign of the estimated standard deviations is irrelevant: interpret them as being positive. | | | | | | | | |

| **Table S6. Checklist for reporting discrete choice experiments in health** | |
| --- | --- |
| **Section**  **Item** | **Page and paragraph** |
| **Purpose and rationale** |  |
| 1. Describe the real-world context and decision-maker that the hypothetical choice context seeks to replicate or inform |  |
| 2. Provide a rationale for using a DCE to answer the research question |  |
| Attributes and levels |  |
| 3. Describe how attributes and levels were derived (e.g. literature review, interviews, focus groups, expert input) |  |
| 4. Provide the final list of attributes and levels |  |
| **Experimental design** |  |
| 5. Report the number of alternatives per choice set and whether they were labelled or unlabelled |  |
| 6. Describe response options (e.g. forced choice, opt-out, status quo) |  |
| 7. Describe the type of experimental design (e.g. orthogonal, D-efficient, Bayesian efficient, partial profile) |  |
| 8. Describe which effects are identified in the design (e.g. main effects, higher order interactions, functional form) |  |
| 9. Describe the number of choice sets, blocks and choice sets per block |  |
| 10. Indicate how the experimental design was obtained (software, catalogue, other) |  |
| **Survey design** |  |
| 11. Provide a sample choice set and the instructions and background information given to respondents (e.g. providing the survey as an appendix) |  |
| 12. Report any randomisation (e.g. choice set order, attribute order, alternative order, framing effects) |  |
| 13. Describe what was checked in piloting (e.g. understanding, respondent burden, timing, wording) |  |
| 14. Report whether information from the pilot was used to update the experimental design (e.g. priors, functional form of attributes) or survey design |  |
| **Sample and data collection** |  |
| 15. Report respondent inclusion/exclusion criteria |  |
| 16. Describe how data were collected (e.g. mail, personal interview, web survey) |  |
| 17. Report the response rate or cooperation rate, if possible |  |
| 18. Report the final sample size and how the sample size was determined |  |
| 19. Describe respondent characteristics and representativeness of target population, if known |  |
| **Econometric analysis** |  |
| 20. Indicate coding of data (e.g. effects, dummy, continuous) including definitions |  |
| 21. Report whether any respondents were removed and why (e.g. suspected fraudulent responses, rationality tests) |  |
| 22. Provide the rationale for model choice (e.g. conditional logit, mixed logit, latent class) and assumptions (e.g. error variance) |  |
| 23. Report model specification |  |
| **Reporting of results** |  |
| 24. Report the model performance, goodness of fit (if comparing models) |  |
| 25. Describe methods used for analysis of model results (e.g. calculation of marginal rate of substitution, attribute relative importance, welfare gain) |  |
| 26. Report measures of precision for the output(s) of interest (e.g. confidence intervals) and how these were derived |  |

| **Table S7. CRC screening preferences of both providers and recipients in different regions** | | | | | | | |
| --- | --- | --- | --- | --- | --- | --- | --- |
| **Attributes and levels** | ***Ref.*** | ***β* / *SD* (95%*CI*)** | | | | | |
|  |  | **Weifang (eastern region)** | | **Tai’an (central region)** | | **Jining (western region)** | |
|  |  | **Recipient** | **Provider** | **Recipient** | **Provider** | **Recipient** | **Provider** |
| **Bowel preparation** |  |  |  |  |  |  |  |
| Yes | No | **-0.157**/0.143  (-0.437, 0.122) | 1.025^***^/0.167  (0.697, 1.352) | 0.499^**^/0.169  (0.167, 0.830) | 0.405^**^/0.128  (0.153, 0.656) | 0.339^*^/0.167  (0.011, 0.668) | 1.032^***^/0.167  (0.705, 1.359) |
| **Screening accuracy** |  |  |  |  |  |  |  |
| 50% | 90% | -1.520^***^/0.236  (-1.984, -1.057) | -1.437^***^/0.221  (-1.871, -1.003) | -0.866^***^/0.198  (-1.254, -0.478) | -0.915^***^/0.180  (-1.267, -0.563) | -1.879^***^/0.254  (-2.377, -1.381) | -0.865^***^/0.163  (-1.186, -0.545) |
| 70% |  | -1.320^***^/0.222  (-1.754, -0.885) | -1.207^***^/0.176  (-1.551, -0.862) | -0.607^**^/0.199  (-0.997, -0.218) | -0.810^***^/0.137  (-1.079, -0.541) | -1.049^***^/0.193  (-1.427, -0.671) | -1.010^***^/0.188  (-1.378, -0.642) |
| **Screening interval** |  |  |  |  |  |  |  |
| Annually | Every ten years | 2.216^***^/0.264  (1.698, 2.734) | 0.052/0.206  (-0.352, 0.456) | 0.769^**^/0.231  (0.316, 1.222) | -0.063/0.184  (-0.423, 0.298) | 1.210^***^/0.253  (0.715, 1.705) | 0.238/0.177  (-0.109, 0.584) |
| Every two years |  | 2.261^***^/0.289  (1.694, 2.828) | 0.848^***^/0.212  (0.431, 1.264) | 1.566^***^/0.239  (1.097, 2.035) | 0.780^***^/0.189  (0.409, 1.151) | 1.033^***^/0.216  (0.609, 1.457) | 0.798^***^/0.188  (0.429, 1.166) |
| Every five years |  | 0.912^***^/0.238  (0.445, 1.380) | 0.074/0.186  (-0.291, 0.438) | 0.425^*^/0.195  (0.042, 0.808) | 0.246/0.167  (-0.081, 0.573) | 0.839^***^/0.218  (0.411, 1.266) | 0.206/0.163  (-0.114, 0.526) |
| **Reduction in CRC-related mortality** |  |  |  |  |  |  |  |
| 10% | 90% | **0.359^*^**/0.180  (0.006, 0.712) | -0.798^***^/0.202  (-1.194, -0.402) | -0.531^*^/0.215  (-0.953, -0.109) | -0.821^***^/0.169  (-1.152, -0.491) | 0.262/0.202  (-0.134, 0.657) | -0.674^***^/0.180  (-1.026, -0.322) |
| 50% |  | -0.166/0.206  (-0.569, 0.237) | -0.998^***^/0.200  (-1.391, -0.606) | -0.754^***^/0.191  (-1.129, -0.379) | -0.881^***^/0.173  (-1.220, -0.542) | -0.700^**^/0.232  (-1.155, -0.245) | -0.815^***^/0.159  (-1.128, -0.503) |
| **Screening costs** |  | -0.002^***^/0.000  （-0.003, -0.002） | -0.001^***^/0.000  (-0.001, -0.001) | -0.000^*^/0.000  (-0.001, -0.000) | -0.001^***^/0.000  (-0.000, -0.000) | -0.002^***^/0.000  (-0.003, -0.001) | **-0.000**/0.000  (-0.000,0.000) |
| **ASC (opt-out option)** |  | -2.043^***^/0.269  (-2.570, -1.515) | -3.790^***^/0.309  (-4.396, -3.185) | -1.596^***^/0.223  (-2.033, -1.159) | -3.504^***^/0.253  (-4.000, -3.008) | -2.237^***^/0.263  (-2.753, -1.722) | -3.293^***^/0.298  (-3.877, -2.708) |
| **Note:** (1) p < 0.05 is considered statistically significant. (* p < 0.05; ** p < 0.01; *** p<0.001) (2) ASC (opt-out option): Represent a specific constant term, the ASC refers to the opt-out option, which was set as the reference alternative. | | | | | | | |

| **Table S8. CRC screening preferences of both providers and recipients with different family histories of cancer** | | | | | |
| --- | --- | --- | --- | --- | --- |
| **Attributes and levels** | ***Ref.*** | ***β* / *SD* (95%*CI*)** | | | |
|  |  | **Family history of cancer (Yes)** | | **Family history of cancer (No)** | |
|  |  | **Recipient** | **Provider** | **Recipient** | **Provider** |
| **Bowel preparation** |  |  |  |  |  |
| Yes | No | 0.448**/0.148  (0.157, 0.738) | 0.581***/0.113  (0.359, 0.802) | **0.133**/0.112  (-0.086, 0.352) | 1.036***/0.133  (0.776, 1.296) |
| **Screening accuracy** |  |  |  |  |  |
| 50% | 90% | -1.111***/0.195  (-1.493, -0.729) | -1.067***/0.156  (-1.373, -0.762) | -1.449***/0.155  (-1.753, -1.144) | -1.104***/0.162  (-1.421, -0.788) |
| 70% |  | -0.988***/0.185  (-1.352, -0.625) | -1.078***/0.145  (-1.361, -0.794) | -1.009***/0.148  (-1.299, -0.719) | -0.992***/0.128  (-1.242, -0.741) |
| **Screening interval** |  |  |  |  |  |
| Annually | Every ten years | 1.361***/0.236  (0.899, 1.823) | **0.169**/0.140  (-0.104, 0.443) | 1.398***/0.169  (1.067, 1.728) | **-0.112**/0.169  (-0.444, 0.219) |
| Every two years |  | 1.571***/0.229  (1.122, 2.021) | 0.742***/0.154  (0.440, 1.044) | 1.601***/0.166  (1.276, 1.927) | 0.905***/0.172  (0.568, 1.241) |
| Every five years |  | 0.741***/0.206  (0.338, 1.144) | **0.248**/0.134  (-0.016, 0.511) | 0.681***/0.146  (0.395, 0.966) | **0.077**/0.143  (-0.204, 0.357) |
| **Reduction in CRC-related mortality** |  |  |  |  |  |
| 10% | 90% | -0.354/0.203  (-0.752, 0.044) | **-0.968*****/0.155  (-1.272, -0.664) | 0.128/0.130  (-0.126, 0.382) | **-0.522*****/0.147  (-0.810, -0.234) |
| 50% |  | -0.409*/0.178  (-0.759, -0.060) | -0.831***/0.140  (-1.105, -0.557) | -0.583***/0.146  (-0.869, -0.298) | -0.944***/0.151  (-1.239, -0.648) |
| **Screening costs** |  | -0.001***/0.000  (-0.001, -0.000) | -0.001***/0.000  (-0.001, -0.000) | -0.002***/0.000  (-0.002, -0.001) | -0.001***/0.000  (-0.001, -0.000) |
| **ASC (opt-out option)** |  | -1.355***/0.218  (-1.783, -0.927) | -4.077***/0.266  (-4.598, -3.556) | -2.091***/0.166  (-2.416, -1.766) | -3.035***/0.201  (-3.429, -2.641) |
| **Note:** (1) p < 0.05 is considered statistically significant. (* p < 0.05; ** p < 0.01; *** p<0.001) (2) ASC (opt-out option): Represent a specific constant term, the ASC refers to the opt-out option, which was set as the reference alternative. | | | | | |

| **Table S9. CRC screening preferences of both providers and recipients of different genders** | | | | | |
| --- | --- | --- | --- | --- | --- |
| **Attributes and levels** | ***Ref.*** | ***β* / *SD* (95%*CI*)** | | | |
|  |  | **Male** | | **Female** | |
|  |  | **Recipient** | **Provider** | **Recipient** | **Provider** |
| **Bowel preparation** |  |  |  |  |  |
| Yes | No | 0.315**/0.114  (0.091, 0.538) | 1.210***/0.246  (0.728, 1.692) | **0.110**/0.122  (-0.128, 0.349) | 0.725***/0.091  (0.546, 0.904) |
| **Screening accuracy** |  |  |  |  |  |
| 50% | 90% | -1.295***/0.174  (-1.636, -0.954) | -1.090***/0.241  (-1.562, -0.618) | -1.199***/0.157  (-1.506, -0.891) | -1.095***/0.127  (-1.344, -0.846) |
| 70% |  | -1.011***/0.157  (-1.319, -0.704) | -1.185***/0.214  (-1.605, -0.764) | -0.817***/0.150  (-1.110, -0.523) | -0.966***/0.105  (-1.171, -0.761) |
| **Screening interval** |  |  |  |  |  |
| Annually | Every ten years | 1.205***/0.198  (0.817, 1.593) | **-0.473**/0.282  (-1.026, 0.079) | 1.355***/0.177  (1.008, 1.701) | **0.200**/0.119  (-0.034, 0.433) |
| Every two years |  | 1.431***/0.192  (1.055, 1.807) | 1.066***/0.285  (0.507, 1.624) | 1.551***/0.171  (1.215, 1.886) | 0.707***/0.118  (0.477, 0.937) |
| Every five years |  | 0.647***/0.157  (0.339, 0.954) | **0.276**/0.227  (-0.169, 0.721) | 0.728***/0.158  (0.419, 1.037) | **0.200**/0.112  (-0.019, 0.419) |
| **Reduction in CRC-related mortality** |  |  |  |  |  |
| 10% | 90% | 0.039/0.150  (-0.256, 0.333) | **-0.722****/0.229  (-1.170, -0.273) | -0.063/0.147  (-0.351, 0.224) | **-0.783*****/0.116  (-1.011, -0.555) |
| 50% |  | -0.363*/0.156  (-0.670, -0.057) | -0.850***/0.234  (-1.309, -0.391) | -0.615***/0.147  (-0.902, -0.327) | -0.895***/0.116  (-1.122, -0.669) |
| **Screening costs** |  | -0.001***/0.000  (-0.002, -0.001) | -0.001***/0.000  (-0.001, -0.000) | -0.001***/0.000  (-0.002, -0.001) | -0.001***/0.000  (-0.001, -0.000) |
| **ASC (opt-out option)** |  | -2.075***/0.192  (-2.452, -1.698) | -3.305***/0.330  (-3.952, -2.659) | -1.585***/0.170  (-1.918, -1.252) | -3.509***/0.182  (-3.866, -3.152) |
| **Note:** (1) p < 0.05 is considered statistically significant. (* p < 0.05; ** p < 0.01; *** p<0.001) (2) ASC (opt-out option): Represent a specific constant term, the ASC refers to the opt-out option, which was set as the reference alternative. | | | | | |
